# Age-Related Differences in Multiple Task Monitoring

**DOI:** 10.1371/journal.pone.0107619

**Published:** 2014-09-12

**Authors:** Ivo Todorov, Fabio Del Missier, Timo Mäntylä

**Affiliations:** 1 Stockholm University, Stockholm, Sweden; 2 University of Trieste, Trieste, Italy; MRC Institute of Hearing Research, United Kingdom

## Abstract

Coordinating multiple tasks with narrow deadlines is particularly challenging for older adults because of age related decline in cognitive control functions. We tested the hypothesis that multiple task performance reflects age- and gender-related differences in executive functioning and spatial ability. Young and older adults completed a multitasking session with four monitoring tasks as well as separate tasks measuring executive functioning and spatial ability. For both age groups, men exceeded women in multitasking, measured as monitoring accuracy. Individual differences in executive functioning and spatial ability were independent predictors of young adults' monitoring accuracy, but only spatial ability was related to sex differences. For older adults, age and executive functioning, but not spatial ability, predicted multitasking performance. These results suggest that executive functions contribute to multiple task performance across the adult life span and that reliance on spatial skills for coordinating deadlines is modulated by age.

## Introduction

Multitasking is a complex and loosely defined construct that covers a wide spectrum of goal-directed activities and time frames (e.g. [1]–[7]). A central aspect of multitasking is that more than one task has to be performed within a limited time frame.

Studies of multitasking have typically focused on dual-task performance, often with highly trained experts, in the fields of aviation or vehicle driving (e.g. [8]–[11]). Other studies have assessed planning and implementation of subgoals in simulated work settings (e.g. [12]) and virtual environments [2], [3].

Since, in its essence, multitasking is about handling multiple goal-directed tasks, it requires temporal integration and monitoring of action sequences (e.g., [13]–[15], [5], [6]). Efficient handling of these complexities requires a monitoring strategy that balances the cost of monitoring against the cost of having inaccurate information about the environment [16]–[18]. In this respect, multitasking has been often associated with cognitive control and time management skills, which have been considered as important predictors of multitasking performance (see e.g. [4], [17]).

Monitoring multiple goals while balancing conflicting costs is particularly challenging for older adults, due to reduced executive control functions (e.g., [19]–[21]). These challenges are present in most everyday activities and they are accentuated in some domains, such as the field of air traffic control (ATC). ATC involves frequent monitoring and coordination of multiple aircrafts under continuously changing conditions [22], [23]. Due to the inherent complexity of the profession, the U. S. Federal Aviation Administration regulates a mandatory retirement at fifty-six years of age [24]. While ATC strongly relies on domain-specific expertise, demands on scheduling and interleaving of multiple tasks have become increasingly prevalent in many daily activities [25], [26]. However, despite their practical importance, individual and age-related differences in multitasking have gained very limited attention in past research.

Studies on age-related and individual-differences in multitasking have not only applied merits, but there are also very good theoretical reasons for carrying out such investigations. Indeed, despite some progresses made in more recent research and the existence of some promising theoretical models [13], [15], [4]–[6] the basic cognitive mechanisms and processes underlying multitasking have still to be properly identified. Research on age-related and individual differences in multitasking can give an important contribution to this endeavor by providing valuable information on the determinants of task performance as happens in other areas of cognition, for instance in memory research (e.g., [27], [28]) and judgment and decision making (e.g., [29]–[31]). Unfortunately, as we previously pointed out, very limited attention has been paid to age and individual differences in the multitasking domain. To fill this gap, the present study focused on individual and age-related differences in what we believe are the most central cognitive aspects underlying multitasking performance. Following our earlier work [4], [32], [33], see also [13], [12], [6] we posit that individual and age-related differences in multitasking reflect both domain-specific differences (i.e., how well an individual can perform a component task in isolation) and higher-level differences (i.e., how well an individual can coordinate component tasks, independent of task-specific skills and experiences). More specifically, the starting point of this study was the hypothesis that multiple task performance reflects individual differences in both executive functioning and spatial ability.

Several patient studies and experimental findings suggest that children and adults with executive dysfunctions have great difficulties in handling multiple tasks (see [34], [35] for overviews). Extending these findings, Mäntylä (2013) asked participants to complete a multitasking session with four gender-fair monitoring tasks as well as separate tasks of executive functioning and spatial ability. The tasks were considered gender-fair as previous studies using similar, yet simpler, methodology have not found sex differences at the level of single task performance [36], [37]. A central finding of the Mäntylä (2013) study was that both executive functioning and spatial ability contribute to multiple task performance in young adults. Individual differences in executive functioning (working memory updating) and spatial ability (mental rotation) were independent predictors of multiple task monitoring, but only spatial ability mediated sex differences in multitasking. Inconsistent with popular beliefs and media reports, the findings of the study showed that men exceeded women in multitaskingfi. Furthermore, menstrual changes accentuated these effects, in that sex differences in multitasking (and spatial ability) were observed between males and females in the luteal, but not in the menstrual, phase of the cycle.

Mäntylä (2013; see also [32]) interpreted these findings in terms of a spatiotemporal hypothesis of multitasking. A central assumption of the hypothesis is that most goal-directed tasks, including multitasking, are temporal in that scheduling, monitoring and task interleaving take place on a time scale, and that coordinating multiple goals and deadlines requires a high degree of cognitive control. One way to handle these complexities might be to represent the temporal pattern of deadlines and task goals in spatial terms.

As a support for the spatiotemporal hypothesis of multitasking, evidence from psychophysical experiments [38], [39] and psycholinguistic studies [40]–[42] suggest that adults and children [43], [44] often rely on spatial representations when processing temporal information (see also [45], [46] for overviews). Consistent with the findings of Mäntylä (2013), an extension of this “time-in-space” notion is that individuals with efficient spatial abilities are also better multitaskers than individuals with less developed spatial skills.

To extend the generality of this research, the aim of the present study was to examine age- and gender-related differences in multiple task performance while considering individual differences in executive functioning and spatial ability in a population-based sample of older adults. Mäntylä's (2013) findings suggest that sex differences in multitasking reflect sex-hormone related variability in multitasking, in that the luteal phase of the female menstrual cycle (with increased levels of estrogen) is associated with reduced spatial ability and multitasking performance. One implication of this hypothesis is that age-related differences in multitasking would be primarily mediated by executive functioning and that differences in spatial ability should have a reduced effect when the overall level and (sex-hormone related) variability in spatial ability decrease with advancing age.

Another implication of this hypothesis is that sex differences in multiple task performance would be expected in young adults, but reduced or even eliminated in (postmenopausal) older adults. Alternatively, considering that several aging studies suggest a male superiority in spatial ability (e.g., [47]–[50]), sex differences in multitasking might also be observed in old adults, possibly due to socio-cultural, rather than hormonal, influences on spatial processing [51], [52].

We examined these hypotheses under experimental conditions that emphasized the more abstract characteristics of multitasking while attempting to minimize individual and gender-related differences in domain-specific skills. Specifically, younger and older adults completed a multitasking session involving four (age- and gender-fair) component tasks. Three of these tasks required monitoring multiple deadlines within a limited time frame, and the fourth (background) task involved identification of names (see below, for details). In addition to the 20-min multitasking session involving the four component tasks, participants completed separate tasks of executive functioning (the matrix-monitoring task, [53]) and spatial ability (the Vandenberg and Kuse Mental Rotation Test; [54]).

## Methods

### Participants

Eighty adults, with an equal number of males and females, participated in the study in return for partial course credit or payment. Young participants were university undergraduates between 20 and 38 years of age (mean age  = 25.80). Older adults were between 63 and 73 years (mean age  = 67.16) and were recruited by random sampling via the Swedish population registry. Specifically, a large majority of the older adults were recruited from of a large-scale study on aging, memory and dementia (see Nilsson et al., 1997, for further details concerning sampling and inclusion criteria). There were no sex differences in education (mean  = 12.67 years) or Mini Mental State Examination (mean  = 28.08, range  = 24 to 30). Pretest interviews indicated that none of the older participants were computer gamers, although a majority of them (72%) reported to be experienced computer users.

### Ethics Statement

This research was conducted following the ethical guidelines of the American Psychological Association and participants provided written informed consent prior to participating in the study. The older adults were recruited from the Betula study project, which is approved by the regional Medical Ethical Committee at Umeå University. For the younger adults, according to the ethical guidelines established by the Swedish Research Council, as well as the internal regulations of the Department of Psychology, the experimental design required signed informed consent, which was collected from all participants.

### Materials

Multitasking was assessed with a computerized task, comprising four component tasks (three different counter tasks) and a concurrent name-back. In the counter tasks, digital counters were displayed on the computer screen. Participants pressed spacebar whenever one of the counters showed a target reading, which was defined by a simple rule (see the Procedure section). Each counter was monitored by pressing a designated key, whereupon the corresponding counter appeared for 2 s. To create a multitasking situation that required monitoring of separate component tasks, the counters ran at different rates (3.60 s, 2.72 s, and 2.40 s per item, respectively). The score on the counter task, which was used as a measure of multitasking performance, was calculated as the total number of correct key presses across all three counters (maximum number of correct responses  = 72). In the name-back task, a series of common Swedish first names were presented above the three counters, at the rate of 2 s per name. Participants were instructed to press a mouse key when a target name that was the same as the name presented three steps back, was detected. The stimuli comprised 40 targets (20 male names and 20 female names) and 360 nontarget names. Nontargets were not repeated in the stimulus list. Number of hits and false alarms were the dependent measures for the name-back task. As previously mentioned, performance on the three counters was used as the measure of multitasking. We also computed a combined score of the counter-task and name-back task performance and this overall measure showed the same pattern as the score on the counter task.

Spatial ability was assessed with pen and paper version of the Vandenberg and Kuse Mental Rotation Test (Peters et al., 1995). After receiving written instructions and completing four practice problems, participants were given 3 min to complete one set of problems, followed by a short break, after which they completed another set. The dependent measure of this task was number of correct responses (maximum correct  = 24 for both sets).

**Figure 1:**
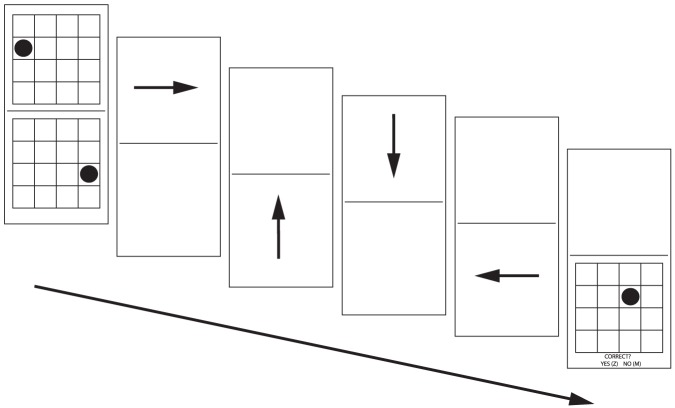
A graphical representation of the Matrix Monitoring task. Each rectangle, beginning from left to right, represents a separate step of the task. In each trial, the participant is first presented with two matrices (upper or lower), each containing a dot (leftmost panel). In the following four steps, arrows appearing in the corresponding part of the screen indicate the movement of the respective dots. After all arrows have been presented, a single, randomly chosen matrix reappears, with the dot in a different location (rightmost panel), and the participant has to decide whether this new position of the dot corresponds to the final position indicated by the sequence of arrows previously presented for that matrix.

Executive functioning was assessed with the matrix-monitoring task, which measures the ability to update working memory representations (Salthouse et al., 2003). In this task, two 4×4 square matrices (10×10 cm) were concurrently displayed in the middle of a 20 inch LCD screen. Separated by a horizontal split line, one matrix was presented in the upper part of the screen and the other one in the lower part. During each trial a black dot was initially presented at a random coordinate within each matrix (see Figure 1). The two matrices with the respective dots were displayed for 3 s, after which they were replaced by a series of four horizontal and vertical arrows alternating in the upper or in the lower part of the screen (see Figure 1), indicating the sequential movement of each dot within its respective matrix. The participant had to imagine the movement of each dot within its respective matrix as indicated by the direction of each arrow, updating the position of the dot within his/her mental representation after the presentation of each arrow. Displaying one arrow at a time and alternating them above or below the split line, at the rate of 1 s per slide illustrated movement of the dot in the upper or in lower matrix, respectively. More specifically, for each trial, the movement of each dot from its initial position in the matrix was indicated by one horizontal arrow and one vertical arrow. After the presentation of the arrows, one of the two matrices reappeared (either the upper one or the lower one, according to a random selection) with the dot repositioned in one of its cells. Then, the participant had to decide whether this new position of the dot corresponded to the final position indicated by the sequence of arrows previously presented for that particular matrix. The main task comprised twelve trials: the upper matrix reappeared in the final step of six trials, while the lower matrix reappeared in the final step of the other six trials. Performance was assessed with the number of correct responses.

### Procedure

Each participant was tested individually during a 45-min session. After completing a background questionnaire, half of the participants completed the Mental Rotation Test, followed by the multitasking session and the matrix-monitoring task. The remaining participants completed the tasks in the opposite order. In the counter tasks, participants monitored each counter for target readings, which were defined by three rules. Specifically, the experimenter instructed participants to press the space bar when the last two digits of Counter 1 were a multiple of 11, when the last two digits of Counter 2 were a multiple of 20, and when the last two digits of Counter 3 were a multiple of 25. The displays for the three counters were in different colors (green for Counter 1, blue for Counter 2, and red for Counter 3). Participants could view the reading of each counter whenever they wanted by pressing a key in the same color, after which the counter would be visible for 2 s. For young participants, responses were considered correct if they were within 1 digit of the target (e.g., 19, 20, and 21 would be considered correct responses if the target was 20). For old participants responses were scored as correct within 2 digits of the target (more strict scoring increased the age effect favoring young adults, but did not have any other effects).

For the name-back task, participants were instructed to press a mouse key whenever the name on the screen was the same as the one presented four names earlier in the sequence. Participants were informed that the three counter tasks and the name-back task were equally important and that the counters were running at different rates. After a 2-min practice session with the four component tasks, one half of the participants completed the 20-min multitasking session first, followed by the Mental Rotation Test or matrix-monitoring tasks, and vice versa for the other half.

## Results

Two old participants did not complete the whole test battery, and thus their data were not included the analysis. The correlation matrix summarized in Table 1 shows highly significant age effects in multitasking, based on mean accuracy for the whole sample (upper diagonal) and separately for the old adults (lower diagonal). Consistent with earlier work, significant age effects were also observed in spatial ability (Mental Rotation Test) and executive functioning (matrix monitoring). Furthermore, young participants with good performance in the counter tasks showed higher scores on the Mental Rotation Test and matrix-monitoring tasks than less efficient multitaskers. For old adults, only executive functioning correlated with multitasking performance. It should also be noted that better educated older adults were more efficient multitaskers than less educated individuals (even after controlling for age). Separate analyses of the name-back data showed a similar pattern of correlations as the counter accuracy data.

**Table 1:** Pearson Correlations for Age. Multitasking. Spatial Ability, and Executive Functioning.

Measure	Age	Multitasking	MRT	Matrix	Education
Age	-	−.65**	−.64**	−.41**	−.32*
Multitasking	−.45**	-	.57**	.55**	.41**
MRT	−.40*	.24	-	.42**	−.01
Matrix monitoring	−.32*	.50**	.39*	-	.27
Education	−.32*	.41**	−.01	.27	-

*p*< .05.

** *p*< .01.

MRT  =  Mental Rotation Test.

Upper diagonal  =  the whole sample (*N* = 78).

Lower diagonal  =  old adults (*n* = 38).

**Figure 2:**
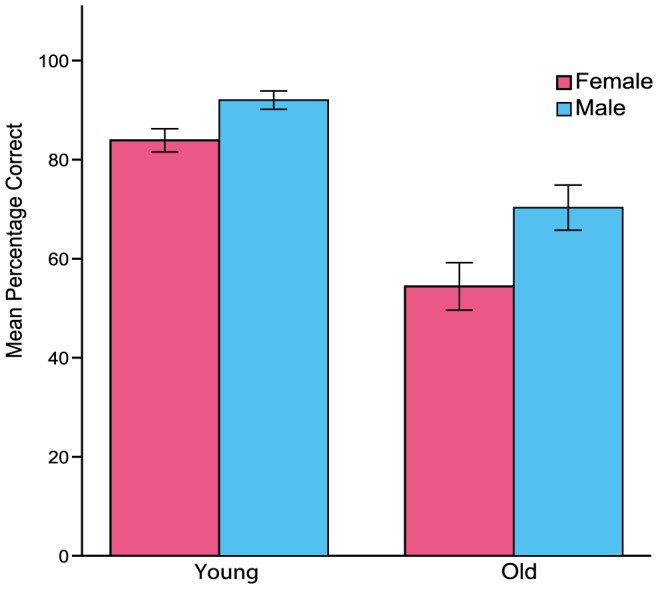
Accuracy in the counter task as a function of age and gender. Error bars denote the standard error of the mean.

Figure 2 shows counter accuracy as a function of age and gender. Consistent with the correlation analysis, young adults (mean  =  .88) were more accurate than older adults (mean  =  .63), *F*(1, 74)  = 47.44, *MSe*  = 0.03, *η^2^* =  .39, *p* =  .001. Furthermore, as shown in Figure 2, males (mean  =  .82) outperformed females (mean  =  .70), *F*(1, 74)  = 9.89, MSe  = 0.03, *η^2^* =  .12, *p* =  .001, with similar sex differences for both age groups (*F*<1). A separate ANCOVA on the counter accuracy data yielded the same pattern of results when monitoring frequency was included as a covariate in the analysis. The name-back data, measured in terms of hits minus false alarms, showed no other effects than a significant gender difference favoring males, *F*(1, 74)  = 3.77, *MSe*  = 830.61, *η^2^* =  .05, *p*< .05. Thus, the observed age and sex differences in counter accuracy were not due to a trade-off between accuracy in the counter tasks and in the background task, given that males outperformed females also in the name-back task. Furthermore, as shown in Table 2, the monitoring frequency data showed a significant age effect, *F*(1, 73)  = 43.44, MSe  = 91.34, *η^2^* =  .34, *p* =  .001, whereas the main effect of gender and its interaction with age were nonsignificant (*ps* > .22).

**Table 2:** Mental Rotation, Executive Functioning, and Monitoring Frequency Data as a Function of Age and Sex with Standard Deviations in Parenthesis.

Group	Monitoring Frequency	Mental Rotation	Executive Functioning
Old Female	20.34 (9.19)	.16 (.13)	.65 (.13)
Old Male	20.66 (8.46)	.21 (.13)	.69 (.15)
Young Female	31.68 (8.87)	.34 (.13)	.76 (.12)
Young Male	36.83 (12.45)	.47 (.16)	.79 (.16)

Note. Monitoring frequency  =  number of counter checks/min.

Executive functioning  =  proportion correct in the matrix monitoring task.

Table 2 summarizes the matrix-monitoring and Mental Rotation Test data as a function of age and sex. An ANOVA on the matrix-monitoring data showed no other effects than a main effect of age, *F*(1, 74)  = 11.95, *MSe*  = 0.02, *η^2^* =  .14, *p* =  .001, favoring young adults (mean  =  .78) over old adults (mean  =  .67). An ANOVA on the Mental Rotation Test data showed significant main effects of age, *F*(1, 74)  = 52.46, *MSe*  = 0.02, *η^2^* =  .42, *p* =  .001, and gender, *F*(1, 74)  = 8.15, *MSe*  = 0.02, *η^2^*  =  .10, *p* =  .006. No other significant effects were observed.

A multiple regression analysis on these data indicated that age (*Beta*  = −.38, *p*< .02), sex (*Beta*  =  .34, *p*< .02), and matrix monitoring (*Beta*  =  .37, *p*< .01), but not Mental Rotation Test (*Beta*  = −.13, *p*< .40), were significant predictors of multitasking performance, *R* =  .76, *F*(4, 73)  = 25.52, *MSe*  =  .02, *p*< .01. Separate analysis of the old adults' data showed a similar pattern in that age (*Beta*  = −.49, *p*< .01), sex (*Beta*  =  .24, *p*< .01), and matrix monitoring (*Beta*  =  .29, *p*< .01), but not Mental Rotation Test (*Beta*  =  .08, *p*< .50), were significant predictors of multitasking performance, *R* =  .67, *F*(4, 33)  = 6.78, *MSe*  =  .03, *p*< .01. However, young adults' data showed that both Mental Rotation Test (*Beta*  =  .35, *p*< .02) and matrix monitoring (*Beta*  =  .29, *p*< .05) were significant predictors of multitasking performance, *R* =  .67, *F*(4, 35)  = 7.02, *MSe*  = 0.01, *p*< .01, while neither age nor sex were significant predictors in this analysis.

We extended these analyses by computing partial correlations between counter accuracy and mental rotation (while controlling for matrix monitoring) and between counter accuracy and matrix monitoring (while controlling for mental rotation), separately for the two age groups. Consistent with the regression analyses, partial correlation between counter accuracy and matrix monitoring was significant both for young adults (*r* =  .38, *p*< .02) and old adults (*r* =  .45, *p*< .01). The partial correlations for counter accuracy and mental rotation showed a different pattern in that the association was highly significant for young adults even after controlling for individual differences in executive functioning (*r* =  .51, *p*< .01). By contrast, this correlation was not significant for old adults (*r* =  .05), suggesting that individual differences in spatial ability did not significantly contribute to old adults' multitasking performance.

## Discussion

The starting point of this study was our earlier findings suggesting that multiple task performance is mediated by individual differences in executive functioning and spatial ability, and that sex differences in young adults' multitasking performance reflect sex-hormone related variability in spatial ability. In the present study, we examined the generality of these findings under the conditions of normal aging in which the overall levels of executive functioning and spatial ability are reduced and hormone-related menstrual effects are eliminated.

The main findings partially supported our hypotheses in that multitasking performance reflected individual differences in both executive functioning and spatial ability, as measured by the matrix-monitoring task and the Mental Rotation Test. Consistent with past research, participants with efficient executive functions were better multitaskers than those with less efficient control functions (even after controlling for spatial ability) and this conclusion was age-invariant. This result, which was observed for the whole sample of adults and separately for the two age groups, was consistent with previous studies showing age effects in dual-task performance (e. g. [55], [56], see also [57] for an overview). However, in our study, these effects were not limited to a contrast between old adults and young (undergraduate) adults, but were also observed within the 10-year age range of the old adults' sample. This finding suggests that multiple task monitoring, and its postulated component processes, executive functioning and spatial ability, are age sensitive.

In addition to executive functioning, multitasking performance was related to individual differences in (younger adults') Mental Rotation Test performance. Thus, good spatial ability was associated with efficient multitasking. This result was observed even after controlling for individual differences in executive functioning, replicating the findings of Mäntylä (2013) and supporting our spatiotemporal hypothesis of multitasking. However, this effect was age-specific in that individual differences in spatial ability did not significantly contribute to old adults' multitasking performance. These results suggest that executive functioning contributes to multiple task performance and that reliance on spatial processes for coordinating deadlines is reduced with advancing age.

The third main finding of the study was that both age groups showed systematic sex differences in multitasking favoring men. This finding extended Mäntylä (2013) results to a population-based sample of older adults. Also in line with that study, sex differences in young adults' multitasking were related to spatial ability, even after controlling for individual differences in executive functioning.

However, in contrast to young adults, old adults' multitasking performance was not related to spatial ability. Instead, our analyses suggest a direct link between gender and multitasking performance even after controlling for individual differences in executive functioning and spatial ability.

Consistent with earlier work on young and old adults (e.g., [47]–[50]), men outperformed women in spatial ability, as measured by the Mental Rotation Test. However, in contrast to the young adults' data, the male superiority in old adults' multitasking performance was not related to spatial ability, as measured with the Mental Rotation Test. The finding that individual differences in spatial ability contributed to young (but not old) adults' multitasking performance might be related to age differences in overall levels of spatial ability. Compared to young adults, both groups of older adults showed rather low levels of performance (M =  .15 for old females), which might have zeroed the effects of Mental Rotation Test in older adults' multitasking performance by shrinking individual differences in spatial abilities. However, it should be noted that the Mental Rotation Test data do not support this potential explanation as the two age (and gender) groups showed rather similar variability in Mental Rotation Test performance and a significant correlation between age and Mental Rotation Test is apparent even in the older adults' data (see Tables 1 and 2).

Thus, the observed sex differences in older adults' multitasking performance might reflect the influence of other factors, such as different aspects of spatial processing than the ones measured by Mental Rotation Test, or nonspatial, age-related differences. The former possibility implies that older adults may rely on different [58] and possibly less demanding spatial strategies than young adults, in order to carry out multitasking, and that individual differences in these types of spatial processes are not well captured by the Mental Rotation Test. As a support for this notion of change of spatial strategies with age, several studies suggest that aged rodents and humans more likely prefer less demanding egocentric (route-based) strategies than allocentric (Euclidean) strategies [59]–[62]. For example, Rodgers et al. (2012) examined age differences in human navigation strategies, and found that older adults preferred an (extrahippocampal) egocentric strategy, whereas younger adults were equally distributed between egocentric and allocentric preferences. Nonspatial factors can also contribute to explain sex differences in older adults' multitasking. It has been shown that numerical skills may support performance in the counter task in addition to executive functioning and spatial abilities [33] and sex differences in these skills may be enhanced by cohort-related effects (e.g. [63]). Future studies may include measures of numerical skills and different measures of spatial ability in order to investigate these potential explanations.

Taken together, the present findings support the hypothesis that individual differences in multitasking reflect independent contributions of executive functioning and spatial ability (as measured by the Mental Rotation Test) in younger adults. Older adults' performance is possibly supported by executive functioning in the same vein, but the influence of different aspects of spatial skills or nonspatial age-related differences needs to be assumed in order to explain the observed sex differences favoring older males vs. older females. An interesting avenue for future work would be to examine individual differences in multiple task performance in relation to other components and mediators of cognitive control and spatial ability
